# The Sound of Emotional Prosody: Nearly 3 Decades of Research and Future Directions

**DOI:** 10.1177/17456916231217722

**Published:** 2024-01-17

**Authors:** Pauline Larrouy-Maestri, David Poeppel, Marc D. Pell

**Affiliations:** 1 Max Planck Institute for Empirical Aesthetics, Frankfurt, Germany; 2 School of Communication Sciences and Disorders, McGill University; 3 Max Planck-NYU Center for Language, Music, and Emotion, New York, New York; 4 Department of Psychology and Center for Neural Science, New York University; 5 Ernst Strüngmann Institute for Neuroscience, Frankfurt, Germany; 6 Centre for Research on Brain, Language, and Music, Montreal, Quebec, Canada

**Keywords:** affective science, emotion, perception, acoustics, human communication

## Abstract

Emotional voices attract considerable attention. A search on any browser using “emotional prosody” as a key phrase leads to more than a million entries. Such interest is evident in the scientific literature as well; readers are reminded in the introductory paragraphs of countless articles of the great importance of prosody and that listeners easily infer the emotional state of speakers through acoustic information. However, despite decades of research on this topic and important achievements, the mapping between acoustics and emotional states is still unclear. In this article, we chart the rich literature on emotional prosody for both newcomers to the field and researchers seeking updates. We also summarize problems revealed by a sample of the literature of the last decades and propose concrete research directions for addressing them, ultimately to satisfy the need for more mechanistic knowledge of emotional prosody.

Prosody—the sound properties of vocal expressions—conveys linguistic as well as paralinguistic information, such as a speakers’ intention (for the case of irony, see Larrouy-Maestri et al., 2023a) and a speakers’ emotional state (Banse & Scherer, 1996). Prosody is thus a crucial tool for human communication.^1^ When it comes to the communication of emotions, a minimal correspondence between the acoustics properties of the signal and the production/perception of a certain emotional or affective state is assumed.^2^ For instance, an influential model of emotion expression and perception proposed by Bänziger et al. (2015), based on Brunswik’s lens model and adapted from Scherer (2013a), distinguishes distal information (i.e., internal state of the speaker as estimated by acoustic analysis of their voice) and proximal information (i.e., listeners’ perception). It addresses both the encoding and decoding processes involved in the vocal communication of emotions in terms of acoustic cues (for an introduction, see also Kamiloğlu & Sauter, 2021). However, the mapping between acoustic information and emotions, or what can be called the *sound of emotional prosody*, remains poorly defined. In the core of this article, we describe progress (and limits) in the search for the sound of emotional prosody and highlight ways to address current challenges.

As a prelude, and to convince skeptical readers about the relevance of emotional prosody to the social, natural, and computational sciences, we outline three of the (many) domains that will benefit from a deeper understanding of this topic. First, psychology (and its developmental, cognitive, and social aspects) would obviously profit from scientific advances because emotional prosody plays a central role in language and communication across the life span. On the perception side, it has been shown that we are sensitive to emotional prosody at an early age (e.g., event-related potential data in sleeping neonates; D. Zhang et al., 2014). The ability to correctly interpret affective states (i.e., positive, neutral, and negative) from expressive speech is already efficient around 5 years and improves with age, although with large individual differences (Sauter et al., 2013). On the production side, infants’ vocalizations increase in complexity early on (Wermke et al., 2021), with intonation patterns found in the first months (Snow & Balog, 2002). Children quickly become proficient in using prosody to be understood (reviewed in Esteve-Gibert & Prieto, 2018). Over time, humans become experienced speakers and listeners, using prosody to form and maintain social positions relative to others (Cheng et al., 2016; Fischer & Manstead, 2008), which in turn influences the behavior of communication partners (Bandstra et al., 2011). Importantly, the effective use of emotional prosody is challenged by aging (Lima et al., 2014; Paulmann et al., 2008). Clarifying the life-span development curve (i.e., from emergence to decline) of emotional prosody, its relation to cognitive abilities, and its role in human interactions relies on a proper description of the sound of emotional prosody.

Second, research on the sound of emotional prosody has clinical implications and thus impacts the medical sciences. The use of emotional prosody in typical communicative contexts, although seemingly natural and effortless, reflects a complex array of perceptual, cognitive, and motor functions that can be selectively disrupted. Deficits in the perception and production of emotional prosody have been identified in children using cochlear implants (e.g., Geers et al., 2013), children with autism spectrum disorder (Rosenblau et al., 2017; Yoshimatsu et al., 2016), and children with attention-deficit/hyperactivity disorder (Chronaki et al., 2015). Difficulties can also appear in brain-damaged patients (e.g., Heilman et al., 2004; Pell & Baum, 1997; Van Lancker & Sidtis, 1992) and in adults with clinical conditions such as schizophrenia (Kantrowitz et al., 2015; Pinheiro et al., 2013), dementia of the Alzheimer’s type (Horley et al., 2010), Parkinson’s disease (Ariatti et al., 2008; Pell, 1996), and depression (e.g., Cummins et al., 2015; Kan et al., 2004; Schlipf et al., 2013). Difficulties using emotional prosody can understandably be debilitating and have considerable consequences for these individuals. It is thus necessary to develop precise diagnostic tools, rehabilitation programs, or coping strategies, all of which rely on a more comprehensive and mechanistic understanding of emotional prosody.

Finally, we live in a society in which the place and role of technology undeniably increase.^3^ On the expression side, more and more devices incorporate artificial speech (Robinson & el Kaliouby, 2009) and aim at sounding as “human” as possible (Drahota et al., 2008) to facilitate human–computer interactions. On the recognition side, the objective is to build tools that can adequately capture the emotional state of a speaker.^4^ Numerous applications of automatic emotion-tracking tools (e.g., Alonso et al., 2017; Wang et al., 2015) have already been proposed, for instance, to improve in-car safety systems (Eyben et al., 2010) and to detect stress or frustration or annoyance in speakers’ voices (e.g., Ang et al., 2002; X. Zhang, Wang, et al., 2015; Zhou et al., 2001). Importantly, benefits of these tools are foreseen in pedagogical and medical contexts in which the communication through nonverbal behaviors between pupil/teacher or patient/physician is crucial (e.g., Alexander et al., 2015; Baruch et al., 2016; Dubey et al., 2016; Griol et al., 2014; Persky et al., 2016; Rochman & Amir, 2013). In addition to easing communication, such noninvasive tools appear promising for detecting disorders such as depression (e.g., Alghowinem et al., 2013; Pan et al., 2019) or autism (Asgari et al., 2021) and thus may be of benefit to public health.

## State of the Art on the Sound of Emotional Prosody

Over the years, several attempts have been made to identify the relevant cues or features of emotional prosody (Murray & Arnott, 1993; Scherer, 1986). As summarized in Bänziger et al. (2015), emotional prosody has been examined from two different angles concurrently. Some studies have focused on acoustic aspects (i.e., encoding), whereas others have focused on the recognition of emotions by listeners (i.e., decoding). The number of encoding studies, in particular, has increased dramatically in tandem with technological advances (for a review of early studies, see Juslin & Laukka, 2003).

One major step toward identifying the acoustic characteristics of emotional prosody was attained by Banse and Scherer (1996). Their study represented a dramatic improvement in methods compared with previous work because they analyzed substantially more affective states (*n* = 14) and increased the number of acoustic features (*n* = 29 relative to pitch, spectral, and temporal dimensions). As reported in Table 1, listeners’ recognition of specific emotions could be predicted by different constellations of features. Importantly, using a jackknifing procedure, the authors identified a subset of the 16 best performing features from the initial 29 parameters: four features concerning the fundamental frequency (*f*_0_); one related to *speech rate*, an estimate of *loudness*; and the others related to *vocal quality/timbre*. The work of Banse and Scherer (1996) has inspired years of research on the sound of emotional prosody and became a standard reference article cited by researchers from the computer sciences, social sciences, neurosciences, medical sciences, and the humanities.

**Table 1.** Acoustic Predictors and General Description of Emotion Categories According to Banse and Scherer (1996)

Emotion category	Dimensions				General description
	Pitch	Temporal	Loudness	Timbre	
Hot anger	X			X	High and bright voice with limited pitch fluctuations
Panic fear	X				High-pitched voice with limited fluctuations
Anxiety	X		X		Quiet voice in the middle pitch range with limited pitch fluctuations
Desperation	X	X		X	High and bright voice with limited pitch fluctuations and a slow speech rate
Sadness			X	X	Quiet and thin voice
Elation	X			X	High-pitched voice with some fluctuations
Boredom	X	X	X		Low and quiet voice with slow speech rate
Shame			X		Quiet voice
Pride	X				Low-pitched voice
Contempt	X				Low-pitched voice with some pitch fluctuations

Note: The pitch dimension includes the mean and standard deviation of *f*_0_. The temporal dimension refers to the duration of articulation periods (i.e., the duration of nonsilent periods). Loudness is estimated with the mean energy (mean of the log-transformed microphone voltage). Timbre includes the Hammerberg index (difference between the energy maximum in the 0–2000-Hz frequency band and in the 2000–5000-Hz band), the proportion of voiced energy up to 1000 Hz, and the slope of spectral energy above 1000 Hz. “X” denotes the significant contribution of acoustic dimensions in predicting the categorization for each emotion. Note that the fit of statistical models for happiness, cold anger, interest, and disgust were lower or the specific contribution of features was unclear. These emotional states are not reported here; for a full description, see Banse and Scherer (1996).

**Fig. 1.**
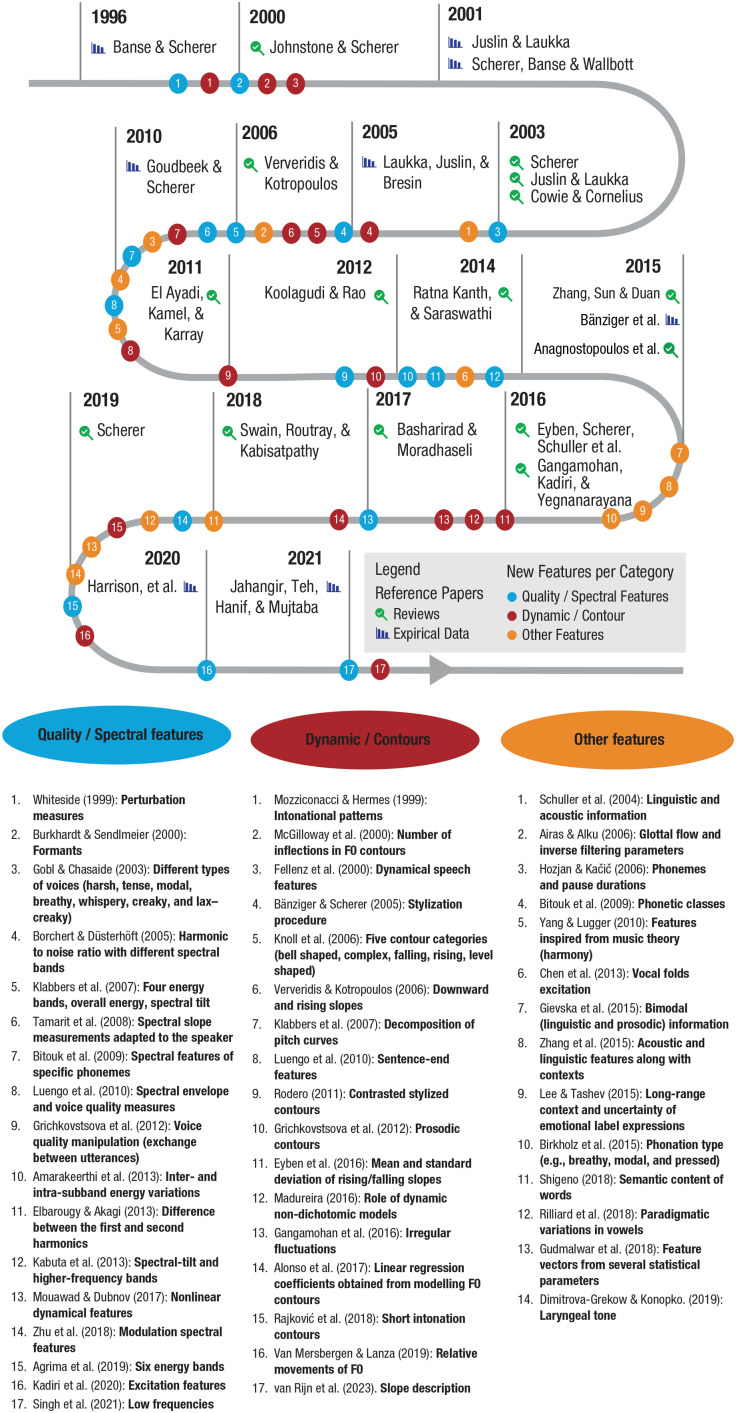
Advances in the acoustic description of emotional prosody since 1996. A limited number of reference articles representing key reviews (magnifying-glass symbol) and highly cited empirical reports (histogram symbol) are especially marked. The numbers represent examples of references using additional features relative to the quality/spectral features (blue), dynamic/contour features (red), as well as other features (orange) over the years.

Since that landmark publication, extensive effort has been made to describe the mapping between acoustics and emotional prosody, in particular by extending the number of acoustic features examined. Figure 1 summarizes a chronological reading of English articles published between 1996 and 2021. By no means exhaustive, this list is grounded in a simple search procedure suited to this interdisciplinary topic: Google Scholar. Indeed, research on emotional prosody can be found in different types of publications that specific tools such as PubMed or Scopus do not necessarily cover. For instance, conference proceedings or patents are the main dissemination technique in engineering, whereas work in the humanities is reported in books and research in the social sciences is described in peer-reviewed journals. Concretely, we used Google Scholar without restriction regarding the format and looked at all entries citing the reference article (Banse & Scherer, 1996). Because of space, we limited the must-read empirical articles to a few references, but a large number of articles with experimental approaches, from both the social sciences and computer sciences, can be found throughout the selection of reviews in Figure 1.

Thanks to technological advances from individual teams and in response to scientific calls for innovations (e.g., INTERSPEECH 2009 Emotion Challenge; Schuller et al., 2009), the number of acoustic features found to be associated with emotional-prosody classification has dramatically grown. Much progress can be observed not only regarding the features quantifying quality/spectral features (see Fig. 1, blue dots) but also in the identification of other features or their interactions with information such as phonemic characteristics or semantic content (see Fig. 1, orange dots). As the number of acoustic features examined increased (Fig. 1), selection/reduction strategies became necessary to identify the most relevant ones (e.g., Dropuljić et al., 2013; Huang et al., 2009; McGilloway et al., 2000; Oudeyer, 2003; Schuller et al., 2004). In an attempt to standardize measurements, Eyben et al. (2016) proposed both a “minimalistic” parameter set (GeMAPS) containing 18 low-level descriptors (relative to frequency, energy/amplitude, and spectrum), some of their derivatives (leading to a total of 56 parameters), and six temporal features. The authors provided a publicly available implementation (with the openSMILE toolkit) to analyze a total of 62 parameters. This set of parameters can then be complemented by additional low-level features, cepstral parameters, as well as dynamic parameters (i.e., the “extended” version of 88 parameters, eGeMAPS), or by any potential additional features relevant for a specific research question or material.

Crucially, the idea of acoustic changes over time or dynamics, which was already found in Fairbanks and Pronovost (1938), has been developed in the last decades (Fig. 1, red dots). Whereas most acoustic features reported in the literature are summary statistics over a unit (word or phrase or sentence), the role of dynamics or pitch changes over time has been repeatedly shown (e.g., Grichkovtsova et al., 2012; Pell & Kotz, 2011; van Rijn et al., 2023), and some attempts have been made to quantify them. For instance, Bänziger and Scherer (2005) and Rodero (2011) marked key points to describe contours through stylization, with tools such as the modeling melody algorithm MOMEL and the International Transcription System for Intonation (INTSINT) developed by Hirst (2005). Another method, proposed by Alonso et al. (2017), consists of modeling the pitch trajectory and interpreting the linear-regression coefficients to describe the pitch height and declination or trend of the pitch contour. More recently, van Rijn et al. (2023) quantified the pitch shape of sentences from existing emotional-prosody corpora in three different ways, including a morphometric method (for previous use in other domains, see M. A. Knoll & Costall, 2015; MacLeod, 1999). Although there is room for improvement of the measures, their study showed that such a method helps capturing the *f*_0_ changes over time and improves the classification of emotions.

To describe the sound of emotional prosody, other approaches that make use of updated statistical methods have also emerged in recent years. Cowen et al. (2019) explored emotion recognition from prosody by analyzing the acoustic correlates of 2,519 speech samples and observed the acoustic features (of speech from 100 actors across five cultures) that tracked 12 dimensions or emotion categories. Their comparison between emotion judgments and acoustic properties across cultures highlighted the relevance of several features, namely duration, pause time, mean *f*_0_, minimum/maximum *f*_0_, first/second/third average formant frequencies, first/third quartiles of the frequency spectrum, spectral centroid, and pitch salience. Another example of the benefit of big data analysis can be found in van Rijn and Larrouy-Maestri (2023), who examined 3,000 min of recordings from various corpora across the globe. Whereas the mapping between acoustic features and emotions varied across corpora, seven acoustic factors named according to the type of features loading on each dimension explained a total variance of 57%: voice quality, loudness, pitch/formants, rhythm/tempo, shimmer, pitch variation, and mel-frequency cepstrum. The factor solutions were quite robust across the most common countries and languages in the data sets. With this elaborated approach, the work of Cowen et al. (2019) and van Rijn and Larrouy-Maestri (2023) confirmed the relevance of key acoustic features in the communication of emotion through prosody but also highlighted the complexity (and opacity) of the mapping between acoustic features and emotion in speech.

## Toward an Updated Definition of Emotional Prosody

Despite the tremendous progress that has occurred since Banse and Scherer (1996), our chronological reading of articles published since then does not lead to a comprehensive and definitive description of the sound of specific emotions. This conclusion was unexpected and probably disappointing (to us and to the reader). As a matter of fact, we observe a lack of consensus between studies, which makes a tentative description particularly speculative. In this section, we discuss possible sources of variability relative to the speech material and to the acoustic characteristics examined. We also reflect on the role of additional factors in the acoustics–emotion mapping. Without being exhaustive, we suggest directions for addressing each point raised in Table 2.

**Table 2.** Suggestions for Next Steps to Investigate the Acoustics–Emotion Mapping

Nonexhaustive sources of variability	Potential next steps
Speech material	
Length and language of the material	Examine the effect of length as well as the role of linguistic/phonological/semantic content of speech material on the acoustics–emotion mapping
Stereotypicality	Investigate the notion of stereotypicality (or caricature) in recorded material, potentially modulated by the type of speaker being recorded; increase variability in the material by recording professional singers who are used to being recorded but not trained in speech production (procedure used in Holz et al., 2022)
Acoustic characteristics	
Choice of the unit size	Identify the minimal size of relevant units, their distinct roles, and their integration in emotion communication through speech
Dynamic aspect	Quantify the dynamic aspect of emotional prosody and its role in emotion communication
Direction and magnitude	Describe the direction and magnitude of acoustic features responsible for the recognition of speakers’ emotional states through prosody, for instance, by investigating listeners’ perceptual thresholds (for methods proposed in the music domain, see Larrouy-Maestri et al., 2019)
Other factors	
Culture	Determine and quantify factors that affect the emotion-acoustic mapping
Emotion type	Extend research to the large range of affective expressions that reflect human emotional states and their nuances
Authenticity	Account for potential overacted material by including speaker or associated variables in statistical analyses (e.g., Zloteanu & Krumhuber, 2021); ensure the perceived authenticity of new data sets by using authenticity ratings instead of (or in addition to) emotion recognition as an inclusion threshold (as proposed by Holz et al., 2022); examine the “humanness” of emotional prosody by examining what makes synthetic voices nonauthentic

### Factors relative to the speech material

The material found in existing data sets ranges from single vowels to full sentences. It has been shown repeatedly that emotions encoded in very short stimuli can be recognized (Paulmann & Kotz, 2008) and that emotion recognition improves as an utterance unfolds or accumulates (Pell & Kotz, 2011; Rigoulot et al., 2013). In addition to differences in terms of the amount of acoustic information available to the listener (Roche et al., 2015), the length of the speech material also affects the nature of acoustic information. For instance, the spectral characteristics of a single /a/ (Waaramaa et al., 2010) will be different from those of a sentence containing various consonants and a large variance among vowels. Note that the material is rarely phonologically balanced, that is, constituted of phonemes of equal frequency of occurrence in natural speech. Therefore, in addition to changing the amount of acoustic information available to the listener, the length of the speech material affects its acoustic characteristics.

In addition to the length of the speech samples, the specific language in which emotional prosody is embedded greatly differs between data sets. Although studying emotional prosody in the context of existing languages enhances the “natural” aspect of the material, it has the disadvantage of allowing an interaction between the emotional prosody and the semantic content of the material (Pell et al., 2011). An alternative could be to use filtered speech (e.g., Bryant & Barrett, 2008) in which the information necessary to access lexical-semantic information is filtered out, thus rendering speech unintelligible (e.g., Flinker et al., 2019). However, removing spectral information might also affect emotional-prosody perception because voice timbre/quality plays a key role (see Fig. 1). Another alternative is to use pseudospeech (e.g., Banse & Scherer, 1996; Pell & Kotz, 2011). However, the creation of Jabberwocky sentences is not random but aims to preserve the rules of specific languages because listeners develop cultural expectations, and “foreign-sounding” material might influence emotional-prosody perception (Liu et al., 2015).

Another important decision for the creation of emotional speech material concerns the recordings and their selection. Ideal data sets should reflect real-life affective utterances produced in typical situations. However, examining the sound of emotional prosody usually requires a certain level of control with regard to the emotional content (i.e., what was specifically intended to be conveyed), the linguistic material (i.e., similar material across emotions), or the speaker (i.e., same performer for different emotions). Therefore, recordings are typically performed in laboratory settings by invited actors or nonactors. It has been shown that the acoustics of play-acted (or posed) recordings differ from those of spontaneous recordings (Jürgens et al., 2011; Juslin et al., 2018). One can assume that actors are able to express themselves in different (imagined) emotional states, thus providing different versions of specific sentences that can be directly compared. In addition, because actors are used to speaking in front of audiences and to being recorded, their stress level (documented as influencing vocal productions; Larrouy-Maestri & Morsomme, 2014; Paulmann et al., 2016) may be lower than that of nonactors in recording situations. Despite these advantages, several shortcomings are potentially associated with actors, such as the overuse of caricatures or stereotypes (Banse & Scherer, 1996; Drolet et al., 2012; Jürgens et al., 2013; Scherer, 2013b), and suggest that nonactors may be more suitable speakers. However, nonactors might be acting as well, without having adequate training to express emotions with plausible variability, and thus may also produce stereotypical stimuli.

In addition to potential factors linked to speakers’ characteristics, the selection of the material itself can play a role in its stereotypicality and thus on the acoustics–emotion mapping. Banse and Scherer (1996) and subsequently several others included recognition tasks performed by small groups of judges or experimenters to discard stimuli that were poorly recognized. Such a procedure is often presented as a validation step. However, by reducing the initial set, this procedure reduces the acoustic variability (e.g., small standard deviations around the mean for each acoustic feature analyzed), which may likewise affect the quality of statistical models and thus bias the acoustic-emotional prosody association observed. In other words, the acoustic content of the material, and probably its stereotypicality as well, depends on the threshold applied to the recognition task for the selection of the speech material to examine.

### Factors relative to the acoustic characteristics

Linguistic elements of different sizes, such as words, phrases, and sentences, are concurrently tracked and temporally integrated (e.g., Ding et al., 2016; Keitel et al., 2018). With regard to emotional prosody, it seems reasonable to hypothesize that units of different size exist and are integrated over time (Jiang et al., 2015; Pell & Kotz, 2011; Waaramaa et al., 2010). Previous research has focused on different units, such as sentences (Chen et al., 2012), segments (Schuller & Rigoll, 2006; Shami & Kamel, 2005), syllables (Agrima et al., 2019), phonemes (Bitouk et al., 2009; Hyun et al., 2010), or selected vowels (Goudbeek et al., 2009), thus supporting the role of acoustic information at these different levels. As a consequence, a realistic acoustics–emotion mapping would require a better understanding of how the acoustic features of speech units of different size potentially interact in longer segments.

In line with the idea of units and supported by empirical evidence (e.g., Grichkovtsova et al., 2012; van Rijn et al., 2023), the dynamics of speech, or how features change over time, greatly matters to listeners. As illustrated in Figure 1 (red dots), several attempts have been made to describe the dynamics of emotional prosody, with symbolic representations (e.g., tones and break indices: Silverman et al., 1992; INTSINT: Hirst, 2005), melodic contours (Cullen et al., 2008; see also Adams, 1976), linear and quadratic functions (Hoicka & Gattis, 2012), or using a morphometric approach (van Rijn et al., 2023). Although such tools and methods are promising, research on emotional prosody could also benefit from descriptors being proposed in adjacent research topics. For instance, pitch trajectories in single words have been quantified when studying trustworthiness perception (Belin et al., 2017), dominance (Ponsot et al., 2018), and certainty/honesty (Goupil et al., 2021). Note that acoustic changes are not limited to pitch but occur in the case of duration and loudness (Goupil et al., 2021) or their combination, as shown in research on stress and prominence perception (for a discussion, see Cole & Shattuck-Hufnagel, 2016).

Finally, the identification of new acoustic features or of their changes over time does not necessarily inform us about the relevance of their direction and magnitude. For instance, low pitch is often associated with a “sad” emotional state relative to the same speaker performing a “happy” stimulus (Banse & Scherer, 1996), but that does not say “how much lower” the voice should be to sound sad. Laukka (2005) presented listeners vocal expressions that were created by morphing prototypical ones along continua (e.g., happiness–sadness or anger–fear). The results of the identification task supported the idea that changes of pitch, intensity, duration, and timbre shift the perception of the emotion. To the best of our knowledge, this promising finding has not been followed by explicit thresholding procedures as proposed in other domains. For instance, in the music domain, Larrouy-Maestri (2018) manipulated the magnitude of a relevant characteristic (i.e., enlarging or compressing pitch intervals within short tonal melodies) and identified thresholds above which performances were no longer perceived as in tune and were interpreted as out of tune. Of course, it is legitimate to wonder whether such approaches can be easily transferred across domains; however, one could argue that, even if there are differences in terms of the content (acoustic features and units of information) or functions between speech and music, there are similarities in terms of the processes underlying their perception such as their categorization (Larrouy-Maestri et al., 2023b). As a consequence, it seems realistic that the manipulation of single acoustic features, as applied in music, could be used to pursue the approach initiated by Laukka (2005) and determine boundaries between categories (i.e., specific emotions) in the case of emotional prosody.

### Other factors affecting the acoustics–emotion mapping

A large number of studies have revealed an in-group advantage for the recognition of speakers’ emotional states through emotional prosody (e.g., Jürgens et al., 2013; Koeda et al., 2013; Laukka et al., 2016; Paulmann & Uskul, 2014; Pell et al., 2009; Riviello & Esposito, 2012; Sauter, 2013; Sauter et al., 2010; Sauter & Scott, 2007; Scherer et al., 2001; Tisljár-Szabó & Pléh, 2014; Waaramaa & Leisiö, 2013). More recently, van Rijn and Larrouy-Maestri (2023) used large-scale Bayesian inference models to quantify the role of culture (country and language of the speaker) on the mapping between acoustic and intended emotions by analyzing a large set of collected speech corpora (more than 3,000 min of emotional speech). Unsurprisingly, culture substantially affected the correspondence between the intended emotional state of the speaker and acoustics of the vocal expressions, which confirms that growing up in a specific cultural and language environment may thus shape the acoustics–emotion association both in production and perception.

Another factor that has been overlooked refers to the granularity of emotions (Kamiloğlu et al., 2020). In the case of positive emotions, research has only recently focused on more than a very limited number of emotions (Sauter & Scott, 2007), and the comparison of the acoustic profiles of different positive emotions revealed differences in the acoustics. For instance, pitch was higher for joy and amusement but lower for lust and admiration, or speech rate was faster for joy and pride but slower for pleasure. Therefore, grouping all positive emotions under a single or limited number of terms (Scherer, 1986) is misleading. More generally, the emotional space in which we communicate is richer than previously studied (Cowen & Keltner, 2021; Keltner, 2019; Keltner et al., 2019), which supports the need for diversification from the six basic emotions studied by Ekman in the 1970s (Ekman & Friesen, 1971; for an extensive description, see Ekman, 1992, 1999). For the study of emotional prosody, such findings encourage researchers to further extend the usual number of emotional states or dimensions (e.g., 14 in Banse & Scherer, 1996; 12 in Cowen et al., 2019) to reach a more realistic view of the range of emotions communicated through prosody.

Last, the role of expression authenticity on the acoustics–emotion mapping is of great interest in a society in which humans are surrounded by synthetic speech. Text-to-speech tools and AI voice generators aim to create intelligible and realistic sounds but, whereas intelligibility is generally accomplished, the voices do not always sound natural and somehow lack “humanity.” In the emotional-prosody literature, whoever is being recorded (actor, nonactor, singer) receives instructions ranging from the direct request of expressing a specific affective state to techniques to induce specific emotions in performers (Bänziger et al., 2012; see also Kamiloğlu et al., 2020), the latter encouraging spontaneity and thus increasing the genuineness of the expressions (Laukka et al., 2013; Lima et al., 2013). It has been suggested that the use of play-acted stimuli affects listeners’ perceptions of the auditory signal (Anikin & Lima, 2017; Drolet et al., 2012; 2014; Lavan et al., 2016). Drolet et al. (2013) observed that the effect of the authenticity of speech (and its potential relation to acoustic features; see Dropuljić et al., 2017) on emotion categorization is reflected early in cortical processing. Whether authenticity is considered in terms of speakers’ ability to express convincing expressions or in terms of listeners’ perception is currently under discussion (Zloteanu & Krumhuber, 2021), but in light of its relevance, it would certainly be an important factor to further investigate.

## Conclusion

Although the existence of an acoustic signature for each possible emotional state is illusory, we (human speakers and listeners) use acoustic cues naturally and seemingly with ease to infer others’ emotional states beyond words. Inspired by the work of Banse and Scherer (1996), the major advances of the last decades have set the stage for a much better understanding of these cues and how they are used in human communication. Nevertheless, the relentless enthusiasm of scientists in various fields has not been sufficient to fully define emotional prosody and clarify the nature of this crucial but complex phenomenon. We hope that by reflecting on potential issues that prevent a consensus about the acoustics–emotion mapping, future research will be in a better position to constructively move this field ahead. We invite the research community to address current challenges and establish a solid foundation for successfully characterizing the sound of emotional prosody, which is located at the nexus of the humanities, computational approaches, and the psychological and brain sciences.

## Transparency

*Action Editor:* Laura A. King

*Editor:* Laura A. King

## Notes

1. Communication can be efficient using other modalities (reading, sign language, Braille, nonverbal communication), but here we focus specifically on speech and spoken language understanding.

2. The strength of the relation between the physical signal and the expression or perception of a specific emotion depends on the theoretical framework; for example, there is a strong acoustic-emotion relation in affect program theories but a more flexible relation in appraisal and constructivist ones. Interestingly, there is empirical evidence supporting both a straightforward mapping (e.g., association between roughness of screams and the expression of fear; see Arnal et al., 2015; for a review on neural response patterning, see Cowen & Keltner, 2021) and a more complex one (see Barrett, 2017; for an example in the visual domain, see Barrett et al., 2019; for a discussion on universality, see Gendron et al., 2018).

3. The humanization of machines, by improving the quality of the expression, and the improvement of recognition systems can also be viewed as threatening. On the recognition side, automatic systems to be used as assistance tools (e.g., to help the elderly with activities around the home) cannot fully substitute necessary social relations with other humans. On the expression side, the ability to mimic emotional states opens the door to manipulating human behavior because emotional prosody influences the behavior of communication partners (Bandstra et al., 2011; Kramer et al., 2014). This has implications in several contexts, such as negotiations (Sinaceur et al., 2015; Wubben et al., 2011), decision-making (Boidron et al., 2016), and politics (Banai et al., 2017; Dietrich, 2014; Marcus et al., 2000). Therefore, to be sensitive to positive versus negative implications, technological advances must be undertaken responsibly and in a well-considered ethical framework.

4. As discussed in Note 2, such a statement can be questioned in the case of constructivist theories of emotions because the relation between an acoustic and a specific emotional state is not straightforward.
